# Interactions of molten salts with cathode products in the FFC Cambridge Process

**DOI:** 10.1007/s12613-020-2202-1

**Published:** 2020-12-30

**Authors:** George Z. Chen

**Affiliations:** 1 Department of Chemical and Environmental Engineering, Faculty of Engineering, University of Nottingham, Nottingham, NG7 2RD UK; 2 Department of Chemical and Environmental Engineering, Faculty of Science and Engineering, University of Nottingham Ningbo China, Ningbo, 315100 China

**Keywords:** FFC Cambridge Process, molten salts, electrolysis, extraction, oxides, sulfides, metals, alloys, reaction mechanisms

## Abstract

Molten salts play multiple important roles in the electrolysis of solid metal compounds, particularly oxides and sulfides, for the extraction of metals or alloys. Some of these roles are positive in assisting the extraction of metals, such as dissolving the oxide or sulfide anions, and transporting them to the anode for discharging, and offering the high temperature to lower the kinetic barrier to break the metal-oxygen or metal-sulfur bond. However, molten salts also have unfavorable effects, including electronic conductivity and significant capability of dissolving oxygen and carbon dioxide gases. In addition, although molten salts are relatively simple in terms of composition, physical properties, and decomposition reactions at inert electrodes, in comparison with aqueous electrolytes, the high temperatures of molten salts may promote unwanted electrode-electrolyte interactions. This article reviews briefly and selectively the research and development of the Fray-Farthing-Chen (FFC) Cambridge Process in the past two decades, focusing on observations, understanding, and solutions of various interactions between molten salts and cathodes at different reduction states, including perovskitization, non-wetting of molten salts on pure metals, carbon contamination of products, formation of oxychlorides and calcium intermetallic compounds, and oxygen transfer from the air to the cathode product mediated by oxide anions in the molten salt.

## 1. Background

In December 1999, the University of Cambridge published an international patent on what is now known widely as the Fray–Farthing–Chen (FFC) Cambridge Process. The process is about electrolytic extraction of metals and alloys directly from their solid compounds in molten salts [1]. Preliminary findings from the test of the FFC Cambridge Process were soon reported in a *Letter to Nature* for the extraction of titanium from titanium dioxide ($TiO_2$) in molten calcium chloride ($CaCl_2$) [2]. The report aroused enthusiastic responses, both positive and critical, from global communities of titanium technologists and researchers [3–5]. In the past two decades, world-wide research and development have confirmed the scientific principle and technical feasibility and flexibility of the process for the extraction of almost all metals listed in the periodic table and their alloys from their respective oxide or sulfide precursors [6–14]. In addition, the FFC Cambridge Process has versatile applications in other fundamental and industrial areas, such as near-net shape manufacturing of metallic artifacts of complex structures, medical implants, oxygen generation on the moon, capture and electrolytic conversion of carbon dioxide ($CO_2$) to various forms of carbon, e.g., carbon nanotubes, carbon monoxide (CO), and hydrocarbon fuels ($C_nH_{2n+2}$, $n < 10$), and rechargeable molten salt metal–air batteries [15–22].

## 2. Basic electrochemistry

The main claim of the FFC Cambridge Process [1] is very general and states a method "for removing a substance (X) from a solid metal, a metal compound or semi-metal compound ($M^1X$) by electrolysis in a fused salt of $M^2Y$ or mixture of salts, which comprises conducting the electrolysis under conditions such that reaction of X rather than $M^2$ deposition occurs at an electrode surface, and that X dissolves in the electrolyte $M^2Y$." This claim makes it very clear that the cathode can be a metal containing another substance (e.g., impurity) or a metal or semi-metal compound including but not limited to metal oxides, which have been mostly studied in the past two decades. It is interesting to point out that later research has demonstrated that the electrolysis of metal sulfides is actually quicker and more efficient than that of metal oxides. However, the invention of FFC Cambridge Process was mostly based on the initial study of electrochemical reduction of $TiO_2$ to Ti metal in molten $CaCl_2$, which is now known as one of the few metal oxides that are most difficult to electro-reduce.

In terms of thermodynamics, data from HSC chemistry 2012 (Outotec), the electrolysis of $TiO_2$ should be fairly feasible, following reactions (1) or (2) and (3), on an inert or carbon anode, respectively. Although reaction (3) seems more feasible than reaction (2) on a carbon anode, the highly spontaneous reaction (4) makes $CO_2$ effectively the main product on the carbon anode. This view can be further explained as follows. With $\lg K = 2\lg\ p_{CO_2} - (2\lg\ p_{CO} + \lg\ p_{O_2}) = 16.1$, it can be established that $p_{CO_2} \gg (p_{CO} + p_{O_2})$ and $(p_{CO_2} + p_{CO} + p_{O_2}) \approx$ atmospheric pressure for bubbles of the mixed anode gases to escape from the anode surface, where $K$ is the reaction equilibrium constant, and $p_{CO_2}$, $p_{CO}$, and $p_{O_2}$represents the partial pressure of the gas $CO_2$, CO, and $O_2$ in the bubble, respectively. Therefore, there should be a negligible amount of CO produced on the carbon anode, particularly when $O_2$ could form on the carbon anode which was experimentally confirmed [20,23–24].

$$TiO_2 = Ti + O_2, \quad \Delta G^{\ominus}\ (900°C) = 732.1\ kJ,\ \Delta E^{\ominus} = 1.897\ V\ \text{(inert anode)} \tag{1}$$

$$TiO_2 + C = Ti + CO_2, \quad \Delta G^{\ominus}\ (900°C) = 336.1\ kJ,\ \Delta E^{\ominus} = 0.871\ V\ \text{(carbon anode)} \tag{2}$$

$$TiO_2 + 2C = Ti + 2CO, \quad \Delta G^{\ominus}\ (900°C) = 300.7\ kJ,\ \Delta E^{\ominus} = 0.779\ V\ \text{(carbon anode)} \tag{3}$$

$$2CO + O_2 = 2CO_2, \quad \Delta G^{\ominus}\ (900°C) = -360.6\ kJ,\ \lg K = 16.1 \tag{4}$$

where $\Delta G^{\ominus}$ and $\Delta E^{\ominus}$ represent the standard Gibbs energy change and the standard cell voltage, respectively.

Reactions (1) to (3) are possible cell reactions, whereas electrode reactions are as follows.

$$\text{Cathode}: TiO_2 + 4e = Ti + 2O^{2-} \tag{5}$$

$$\text{Inert anode}: 2O^{2-} = O_2 + 4e \tag{6}$$

$$\text{Carbon anode}: C + xO^{2-} = CO_x + 2xe \quad (x = 1,\ 2) \tag{7}$$

Reaction (5) represents the electrochemical reduction or deoxidation of $TiO_2$. The same can be written for other metal oxides. Thus, electro-deoxidation and electro-reduction are both used in the literature as scientific terms in place of the FFC Cambridge Process.

Fig. 1(a) illustrates schematically a typical laboratory molten salt electrolysis cell for studying the FFC Cambridge Process. This two-electrode cell is suitable for the electro-reduction of metal oxides and other compounds at the gram scale. The lower part of the steel vessel (or retort) extrudes below and outside the furnace so that it remains at temperatures much lower than that of the molten salt. In this way, any molten salt dripping or condensing at the welding joint between the wall and bottom of the retort will solidify and become noncorrosive [18]. This cell can also be readily modified into a three-electrode cell for fundamental analyses by, for example, cyclic voltammetry and chronoamperometry.

Electrode material selection is crucial to ensure the suc-

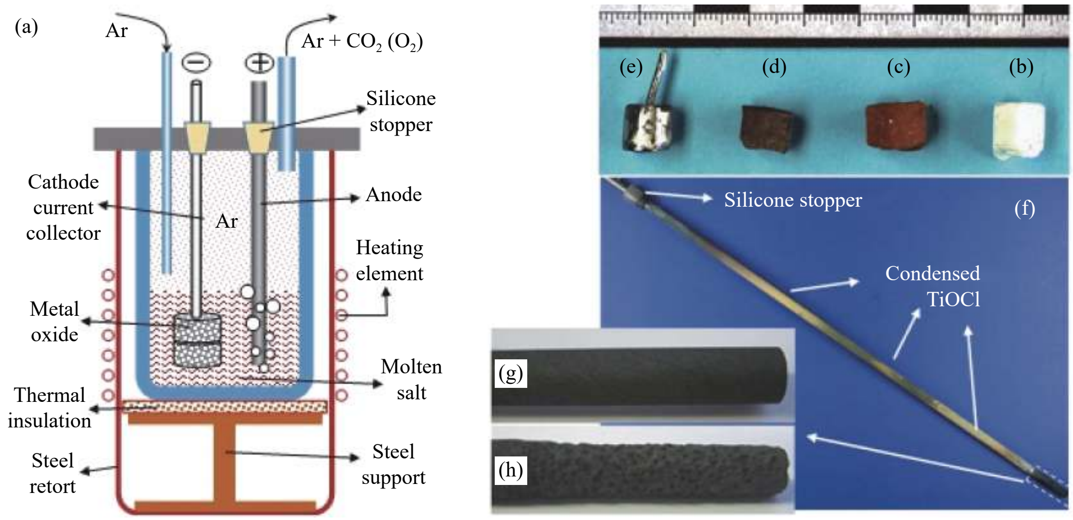

**Fig. 1. (a) Schematic of a molten salt electrolyzer for the laboratory study of the FFC Cambridge Process. The external diameter of the steel retort is typically 15 cm. Digital photographs of slip-cast and sintered pellets of $TiO_2$ (b), mixed $TiO_2$, $Al_2O_3$, and $V_2O_5$ (c), electro-reduced (c, d), surface polished (d, e), and a long graphite rod (circa 1.6 cm in diameter) anode after electrolysis of $TiO_2$ (f); the lower end of the graphite rod before (g) and after 24 h (h) electrolysis.**

cess of the FFC Cambridge Process. For the cathode, Figs. 1(b) and 1(c) show the pellets of $TiO_2$ (white) and mixed $TiO_2$, $Al_2O_3$, and $V_2O_5$ (brown) that were to attach to, and be electro-reduced on the cathode current collector. The products in Figs. 1(d) and 1(e) were the Ti–6Al–4V alloy before and after light polishing, respectively. For the anode, because of their easy availability, ease of shaping, and low cost, commercial graphite products, particularly rods (Fig. 1(f)) and plates, have been commonly used to make the anode or counter electrode in various studies on the FFC Cambridge Process, although the quality of commercial graphite varies significantly. Poor-quality graphite may suffer from the attack of oxidation and gas bubbling, leading to graphite erosion (see the comparison in Figs. 1(g) and 1(h)), and unwanted carbon debris off the anode. The carbon debris can float on or suspend in molten salts, causing electronic conduction to lower the electrolysis efficiency and contamination of the cathode product. More discussions are given later on the issues from using a graphite anode. Glassy carbon can also be used to make the anode or working electrode in studies of molten salts, particularly for fundamental analysis by cyclic voltammetry [25–26]. Similar to graphite, glassy carbon suffers from electrochemical oxidation in the presence of oxide ions and hence is too expensive to use in bulk electrolysis.

In addition, it has been recognised that the discharge (electro-oxidation) of oxide ions ($O^{2-}$) on a carbon electrode suffers from serious kinetic difficulties [25–26]. These complex kinetic steps lead to a fast increase in polarization with increasing the current density on the anode. The kinetics is partly responsible for the practically applied cell voltage for the electrolysis of $TiO_2$ to be at or greater than 3.00 V, in contrast to the thermodynamic predictions of around 1.00 V according to reactions (2) and (3). To minimise such kinetic barriers, the surface area of the graphite anode in contact with the molten salt should be as large as realistically possible. It was found that the anodic polarisation could be reduced by about 1.0 V when the graphite anode surface area was increased by ten times [26]. Alternatively, inert anodes should be considered as an option.

By definition, inert anodes should be inactive and non-consumable, and would work without all the problems resulting from using carbon. Although the potential for anodic formation of $O_2$ is about 1.00 V higher than that for $CO_2$ formation (reactions (1) and (2)), this benefit of using carbon anodes could be largely lost to the above mentioned kinetic polarisation. There are two types of ceramic-based inert anode: ion-blocking and ion-conducting inert anodes (Figs. 2(a) and 2(b), respectively). Several materials have been tested for making ion blocking inert anodes, including tin oxide ($SnO_2$) with or without copper doping, and calcium ruthenate ($CaRuO_3$) with or without titanium substitution [27–28].

Ion-conducting inert anodes are constructed with a membrane of oxide ion conductor, typically yttria-stabilized zirconia (YSZ) in the form a tube with a closed end. One side of the membrane, the wet side, faces the molten salt to connect $O^{2-}$ conduction into the membrane. The other side, or dry side of the membrane can be made in contact with a liquid or solid metal, e.g., tin (Sn) or silver (Ag), with relatively high diffusivity and solubility of atomic oxygen [29–30]. Oxidation of $O^{2-}$ occurs at the YSZ/metal interface, with the produced atomic oxygen dissolving in and diffusing through the metal to the metal/air interface, where the $O_2$ gas forms and escapes. Alternatively, the dry side of the YSZ membrane may be coated with a porous platinum (Pt) paste in which is buried a Pt mesh or wire as the current collector. The oxidation of $O^{2-}$ is catalyzed by Pt at the YSZ/Pt/air three-phase interlines (3PIs) with the produced $O_2$ gas escaping through the pores in the paste into air [31]. The main issues of ceramic-based anodes include their highly temperature-dependent resistivity, thermal cyclability, and related costs.

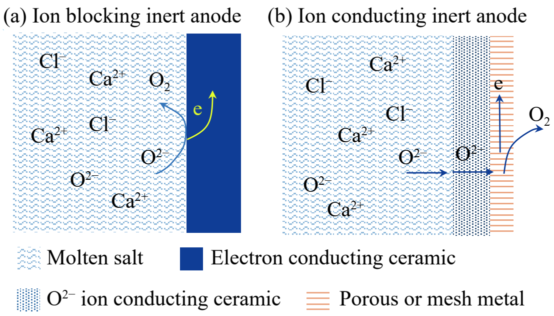

**Fig. 2. Schematic illustrations of the working mechanisms of (a) ion-blocking and (b) ion-conducting inert anodes.**

It is worth mentioning that graphite anodes are almost fully inert when discharging sulfide ions ($S^{2-}$) to the sulfur vapor ($S_2$) [32–33] and chloride ions ($Cl^-$) to the chlorine gas ($Cl_2$) [34–35] in molten chlorides. In electrolytes with high oxide ion activity, such as molten carbonates and mixed oxide melts, refractory metals with a high tendency to form a stable surface oxide layer may also be used as ion-blocking inert anodes [36–38]. However, several metals, e.g., iridium (Ir) and silver (Ag), do not form stable oxides at high temperatures and may behave sufficiently stable as an inert anode in certain molten salts [12,39–40].

## 3. Perovskitization of metal oxides on cathode

In a laboratory cell (Fig. 1(a)), the cell voltage applied for the electrolysis of $TiO_2$ is usually about 3.00 V with a graphite anode, although the thermodynamic prediction from reactions (2) and (3) is less than 1.00 V. Apart from the kinetic difficulties on a carbon anode as mentioned above, cathodic processes also contribute to the observed high cell voltage. First, the high voltage is partly related to the multiple intermediate phases between $TiO_2$ and the Ti metal. At the electrolysis temperature (850 to 950°C), these phases include

$Ti_nO_{2n-1}$ ($n \geq 2$, Magneli phase), $Ti_2O_3$, TiO, $Ti_yO$ ($y$ = 1.5, 2, pseudo-oxides), and solid oxygen solution. Thus, it can be expected that the electro-reduction of $TiO_2$ will pass through each of these phases before reaching the final metallic phase. From available thermodynamic data, the cell voltages needed to decompose TiO and $Ti_3O_2$ with an inert or carbon anode are given in reactions (8) to (11). When the oxygen activity is lower than that in TiO or $Ti_3O_2$, O and Ti combine into a solid solution from which oxygen removal becomes increasingly controlled by O diffusion in Ti, with a secondary effect from the applied cathode potential.

$$2TiO = 2Ti + O_2, \quad \Delta G^{\ominus}\ (900°C) = 860.9\ kJ,\ \Delta E^{\ominus} = 2.231\ V\ \text{(inert anode)} \tag{8}$$

$$Ti_3O_2 = 3Ti + O_2, \quad \Delta G^{\ominus}\ (900°C) = 863.5\ kJ,\ \Delta E^{\ominus} = 2.237\ V\ \text{(inert anode)} \tag{9}$$

$$2TiO + C = 2Ti + CO_2, \quad \Delta G^{\ominus}\ (900°C) = 464.9\ kJ,\ \Delta E^{\ominus} = 1.205\ V\ \text{(carbon anode)} \tag{10}$$

$$Ti_3O_2 + C = 3Ti + CO_2, \quad \Delta G^{\ominus}\ (900°C) = 467.4\ kJ,\ \Delta E^{\ominus} = 1.211\ V\ \text{(carbon anode)} \tag{11}$$

These changes are small compared with those of reactions (1) and (2) and insufficient to account for the experimental observation of 3.00 V. Therefore, kinetics must have played important roles that lead to cathodic polarization. These kinetic difficulties were found to be caused largely by the formation of various perovskites on the cathode during electrolysis, resulting from interactions between the original and partially reduced $TiO_2$ and the calcium dication ($Ca^{2+}$) [7,41–42]. Perovskitization occurs electrochemically and chemically as exemplified below, but TiO and $Ti_3O_2$ have no chemical interaction with $Ca^{2+}$.

$$TiO_2 + 2ze = TiO_{2-z} + zO^{2-} \quad (0 \leq z \leq 0.5) \tag{12}$$

$$mTiO_2 + Ca^{2+} + O^{2-} = CaO\cdot(TiO_2)_m \quad (m \geq 1),\ (CaO\cdot(TiO_2)_m \text{ becomes } CaTiO_3 \text{ at } m = 1) \tag{13}$$

$$mTiO_2 + \delta Ca^{2+} + 2\delta e = Ca_\delta(TiO_2)_m \quad (m/\delta \geq 2),\ (Ca_\delta(TiO_2)_m \text{ becomes } CaTi_2O_4 \text{ at } m/\delta = 2) \tag{14}$$

$$nTiO_{2-z} + Ca^{2+} + O^{2-} = CaO\cdot(TiO_{2-z})_n \quad (n \geq 2),\ (CaO\cdot(TiO_{2-z})_n \text{ becomes } CaTi_2O_4 \text{ at } n = 2,\ z = 0.5) \tag{15}$$

Reactions (13) to (15) add $Ca^{2+}$ to the cathode oxide phase without any oxygen removal, leading to volume expansion of the solid phase, which in turn reduces or eliminates the pore volume in the cathode. As a result, pores for $O^{2-}$ transport in the oxide cathode are partially or fully blocked, resulting in cathodic polarization. Perovskitization also occurs at the cathode of other metal oxides, such as chromium (Cr) and niobium (Nb) oxides [43–44].

Two approaches have been proposed and laboratory demonstrated to avoid or minimize the effect of perovskitization. The first approach uses *ex situ* perovskitization, i.e., reacting $TiO_2$ with CaO or $Ca(OH)_2$ at elevated temperatures (e.g., 1300°C for 5 h) to form a porous $CaTiO_3$ precursor (cylindrical pellet) in air and then using the $CaTiO_3$ precursor as the cathode for electrolysis in molten $CaCl_2$. In this case, perovskitization will not occur at the $CaTiO_3$ cathode during electrolysis, but the cell voltage for electrolysis of $CaTiO_3$ should be slightly higher than that of $TiO_2$, as can be observed by comparing reactions (16) and (2).

$$CaTiO_3 + C = Ti + CaO + CO_2, \quad \Delta G^{\ominus}\ (900°C) = 423.7\ kJ,\ \Delta E^{\ominus} = 1.098\ V\ \text{(carbon anode)} \tag{16}$$

Initial tests of this approach were carried out at 3.20 V and 850°C in molten $CaCl_2$ [42]. The results showed that under the same experimental conditions, the electrolysis of $CaTiO_3$ was almost twice faster as that of $TiO_2$ to achieve Ti products of comparable purities. The higher speed of electrolysis also reflected the faster transportation of $O^{2-}$ in the $CaTiO_3$ cathode than that in the $TiO_2$ cathode. This finding can be explained by the electro-reduction of $CaTiO_3$ removing not only $O^{2-}$ but also $Ca^{2+}$, resulting in increased porosity. This condition is in contrast to that of the electro-reduction of $TiO_2$, whose perovskitization results in the increased volume of the solid phase and hence blockage of the pores in the oxide cathode, impeding the removal of $O^{2-}$ and the whole electrolysis. Continuous $CaTiO_3$ electrolysis via Reaction (16) will lead to the accumulation of CaO in the molten salt. However, one can in principle combine Reactions (16) and (13) to form a closed loop in which CaO is cycled and functions like a "phase change catalyst" to accelerate the electrolysis of $TiO_2$.

The second approach increases the porosity of the $TiO_2$ cathode using the low cost and recyclable $NH_4HCO_3$ as the fugitive porogenic agent. In most previous studies on the FFC Cambridge Process, the $TiO_2$ cathode had usually a porosity of 40% to 50%. Because the molar volumes of $TiO_2$ and $CaTiO_3$ are 18.9 and 34.2 mL/mol, respectively, perovskitization can lead to a volume increase of up to 81%. Obviously, when perovskitization occurs inside the pores of the $TiO_2$ cathode with 40% to 50% porosity, partial blockage of the ion channels is inevitable. Given that perovskitization of the $TiO_2$ cathode proceeds with electro-reduction which follows the 3PI propagation mechanism [45–47], the effect of perovskitization can be possibly bypassed by increasing the $TiO_2$ cathode porosity to 60% to 80%. As a result, the formation rate of TiO and the pseudo-oxide phases surpasses the rate for perovskitization. In other words, the perovskite phase, if formed, is unable to grow in size before further reduction.

This phenomenon was confirmed by experiments in which electro-reduction was most effective when the porosity of the $TiO_2$ precursor was about 68% [48]. At high porosities, the cathode volume increased, extending the path and time for $O^{2-}$ to diffuse out of the porous cathode. Another benefit was that the porous $TiO_2$ cathode prepared from using $NH_4HCO_3$ as the fugitive agent presented a micro-macro-bimodal porosity (Fig. 3(a)). The macropores of over 100 µm in length were left by the evaporation of $NH_4HCO_3$ granules, which were mixed, pressed, and sintered together with the $TiO_2$ powder which alone would only form micropores between the sub-micrometer particles. The sample shown in the SEM image in Fig. 3(a) was fully metallized at 3.20 V in 3 h and contained 0.68wt% O. Continuing the electrolysis at a low voltage of 2.60 V for another 3 h led to a further decrease in the oxygen content to 0.19wt%, whereas the energy consumption was as low as 21.5 kW·h·kg$^{-1}$ for production of Ti. This observation proves a considerably small kinetic influence on removing oxygen from the metallized $TiO_2$ cathode.

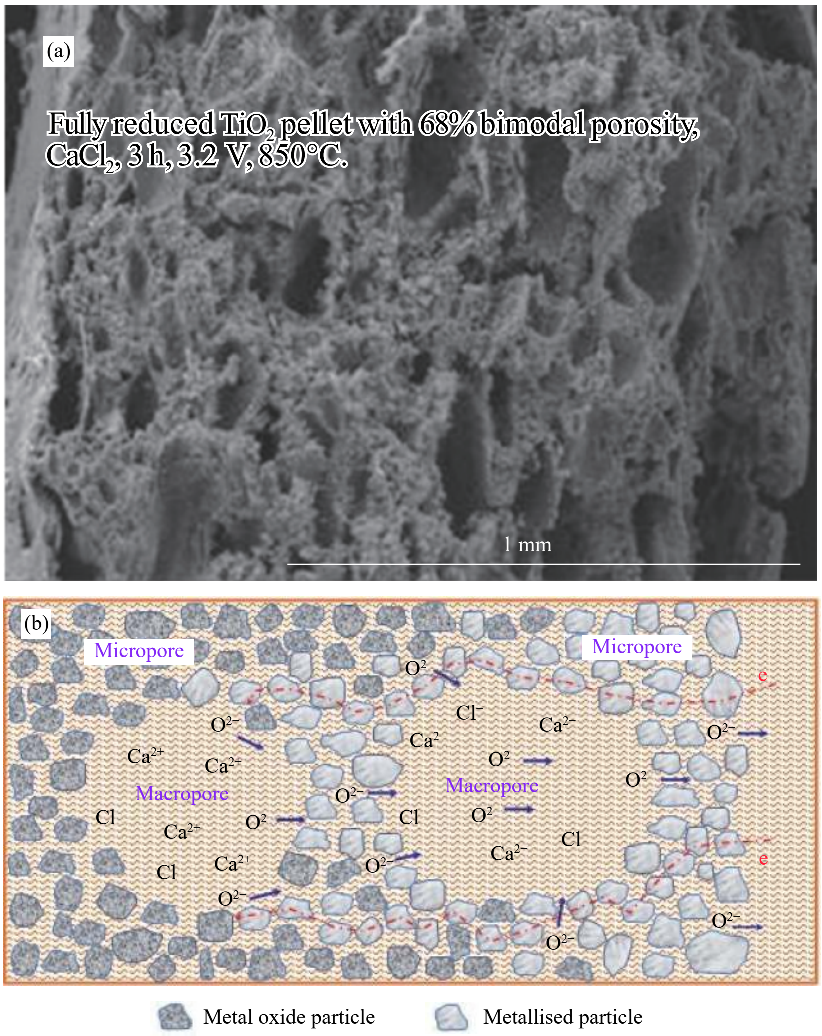

**Fig. 3. (a) Scanning electron microscopy (SEM) image of the micro-macro-bimodal porous structure of a titanium sample that was prepared by electrolysis of the pressed and sintered pellet of mixed $TiO_2$ and $NH_4HCO_3$ powder [48–49]; (b) schematic illustration of $O^{2-}$ transport in the bimodal porous structure. The molten salt in the large pores effectively dilutes the $O^{2-}$ from electroreduction of $TiO_2$ and hence hinders perovskitization.**

Fig. 3(b) further illustrates how the micro-macro-bimodal porous structure of the $TiO_2$ cathode benefits electro-reduction. First, the cathodically produced $O^{2-}$ may participate in either or both competing reactions: perovskitization in the solid phase of the cathode and dissolution into the liquid molten salt. Obviously, dissolution is beneficial to electro-reduction. Both reactions proceed with the assistance of $Ca^{2+}$, whose activity in the molten salt can be assumed to be constant. Thus, the key to prevent perovskitization or keep it short-lived upon formation is to maintain a low $O^{2-}$ activity in the molten salt. Such a goal is difficult, if not impossible, to achieve in the micropores that are present dominantly in the pressed and sintered pellets of $TiO_2$ powder with 40%–50% porosity. With the use of $NH_4HCO_3$ to form the micro-macro-bimodal porous structure, the relatively large amount of molten salt in the macropores can reduce the activity of $O^{2-}$ below what is needed to precipitate CaO, preventing perovskitization.

Second, the transport of $O^{2-}$ in the molten salt of macropores should behave substantially the same as in the bulk electrolyte with the diffusion coefficient in the range of $10^{-5}$ $cm^2/s$, whereas in micropores, the diffusion coefficient of $O^{2-}$ is about $10^{-7}$ $cm^2/s$. Therefore, with the fast ion transportation and minimized effect of perovskitization, electro-reduction occurs rapidly in the micro-macro-bimodal porous $TiO_2$ cathode. The proposed increase of 10% to 20% in cathode porosity may translate to reduced volumetric productivity, which should be well balanced by the benefits in speed increase and energy saving from using a more porous cathode.

## 4. Non-wetting of molten salts on metals from fully electro-reduced cathodes

The world's first 99.8% pure Ti product from electro-reduction of $TiO_2$ in molten $CaCl_2$ showed that the porous oxide precursor transformed into a porous metal (Fig. 4(a)). A technical question asked regarding the FFC Cambridge Process was how to remove the salt that had solidified inside the porous metallic products. Since $CaCl_2$ has a high solubility in water (100 g/mL), and most transition metals and their alloys are relatively stable in water, most, if not all, porous and powdery FFC metal samples reported in the literature were washed in water. Could it be fully effective to remove solidified salts hidden deep inside the pores? Also, because the nodular Ti particles were much larger than the spherical particles in the $TiO_2$ precursor, the growth of Ti particles must have occurred. Therefore, was molten $CaCl_2$ possibly enclosed in the metal in the course of Ti particle growth?

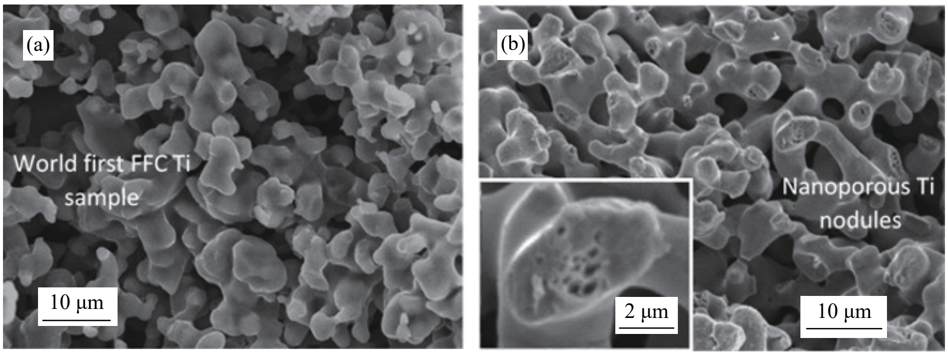

**Fig. 4. SEM images of (a) the first sample of FFC Ti with 99.7% purity (energy-dispersive X-ray spectroscopy (EDX)) produced in Cambridge in late 1997 with a porous structure formed by interconnected micrometer-sized nodules and (b) FFC Ti nodules of which several were broken, revealing nanometer-sized pores (inset) [50].**

Analyses of the FFC Ti samples by SEM and X-ray diffraction (XRD) revealed an interesting behavior. As shown in Fig. 4(b), with careful selection and inspection, nanometer-sized, empty pores were found in the cross section of some relatively large but broken Ti nodules [50]. It could be argued that $CaCl_2$ was removed by washing the sample in water and drying before the SEM examination. However, such nanopores should also exist in most unbroken Ti nodules. If these closed nanopores were filled with $CaCl_2$, the salt should have been detected by XRD, but it was not [42,48]. A hypothesis had attributed the formation of nanopores to the space left after the removal of a large amount of oxygen in the initially formed metallic phases [50]. For example, the pseudo-oxide phase of $Ti_3O_2$ has an oxygen content of 18.2wt%. This rationale is acceptable, but it cannot explain the undetected $CaCl_2$ in the pores of the porous FFC Ti product.

Another explanation came to light when the cause for an incidental observation was considered. In an experiment for the electro-deoxygenation of a Ti foil sample in a Ti crucible, both the foil and crucible were polarized negatively at −3.0 V against a common graphite anode. When electrolysis was near the designated time of completion, the Ti foil was moved forward and backward to drive away a thin layer of floating carbon debris. The manual operation incidentally led to the contact between the foil and crucible. Unexpectedly, the Ti foil stuck to the Ti crucible, and the two were only separated after cooling and washing away the solidified salt and forcing a screwdriver between them with a hammer. Obviously, the Ti foil was welded to the Ti crucible. In the absence of impurities, such as oxide, pure metal-to-metal mixing occurs, resulting in an integral bond. This principle is the foundation of an industrial technique called friction welding. Although friction generates heat and hence high temperatures to promote atom mobility during friction welding, the metals remain solid without melting [51]. In the case of Ti foil and Ti crucible, given that both were negatively polarized for a sufficiently long time, their surfaces must have become oxygen-free and hence were able to weld or bond. However, there was the molten salt between the two Ti surfaces, and why the welding still proceeded can only be explained by the molten salt being non-wetting to pure Ti surfaces.

This understanding of non-wetting of molten salts on a pure metal surface accounts for another observation of the absence or very little of salt in a well electro-reduced and very lightly washed pellet of mixed nickel, manganese, and gallium oxide powders [52]. Later, purposely designed experiments were performed to study the wetting of molten $CaCl_2$ on the surface of terbium (Tb) metal [53]. In the first test, a fully electro-reduced (metallized) porous pellet of $Tb_2O_3$ (converted from $Tb_4O_7$) powder was cooled to room temperature and broken without washing into two halves to reveal the cross section, which was directly analyzed by SEM and EDX. As expected, $CaCl_2$ was only detected on the surface region (labeled A), whereas the contents of Cl and Ca decreased quickly into the metallized pellet (from B to E) (Fig. 5(a)). The SEM image of nodules in Fig. 5(b) was captured from the sandpapered surface of the fully metallized sample after rinsing in dimethyl sulfoxide (DMSO) to remove the debris from grinding. These nodules were very clean, whereas $CaCl_2$ is insoluble in dry DMSO.

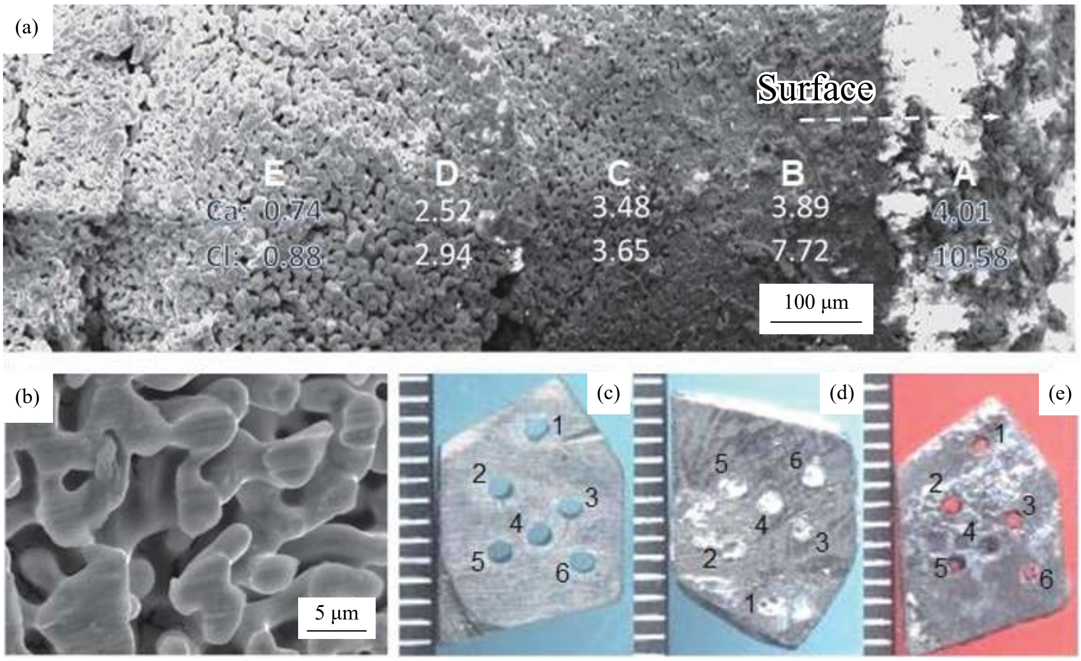

**Fig. 5. SEM images of (a) the cross section of a fully electro-reduced and unwashed porous pellet of $Tb_2O_3$ powder in molten $CaCl_2$ with EDX analysis results indicated for Ca, Cl, and Tb (balancing) and (b) an enlarged view of Tb nodules in the fully electro-reduced $Tb_2O_3$ pellet with several being ground to reveal the non-porous cross section. Digital photographs of a small piece of Tb foil with six drilled holes (c) before and (d) after immersion in molten $CaCl_2$ without electrolysis, showing all holes being filled with solidified salt, and (e) after electrolysis at 3.2 V for 30 min under argon, showing five of the six holes being free of solidified salt [53].**

In another experiment, a small sheet of pure Tb metal was drilled with six small holes (1 mm diameter) and immersed in molten $CaCl_2$ in air. Upon removing the Tb sample from the molten salt, cooling in air, and scraping away the skin of solidified salt, all the six holes were filled with solidified salt. This change is understandable because the Tb metal surface was covered with a thin oxide layer on which the molten salt can wet (Figs. 5(c) and 5(d)). The Tb sheet was then placed back into the molten salt and allowed to electro-reduce against a graphite anode at 3.2 V for 30 min under argon. After electrolysis, the electro-reduced Tb sheet was removed from the molten salt and cooled in air. Five of the six holes were empty (Fig. 5(e)), which must have resulted from the electro-reduced Tb sheet surface being free of oxygen. As a result, the walls of the small holes were unwettable by the molten salt which was driven out of the holes by gravity when the Tb sheet was lifted above the molten salt.

In conclusion, past experimental observations have proven qualitatively and conclusively that molten $CaCl_2$ does not wet the wall surfaces of the internal pores in fully electro-metallized metal oxide cathodes. This finding is theoretically accountable and practically meaningful to the engineering design of the FFC Cambridge Process in terms of separation of the metallic product from solidified molten salt in post-electrolysis processing. This finding also allows the use of organic solvents with a lower but sufficient solubility of the salts and notably lower reactivity to the as-produced metals, particularly rare earth (Re) metals [54].

## 5. Carbon contamination

The use of a carbon anode is common in molten salt electrolysis, as has been the case in most reported studies of the FFC Cambridge Process. Graphite is mostly used, although glassy carbon was also used in several fundamental studies. The advantages of using a graphite anode are basically commercial availability, shaping and processing convenience, thermal and chemical stability in molten salts, and good electronic conductivity. According to reaction (2) and similar ones, the emission of $CO_2$ from the FFC Cambridge Process using a carbon anode is inevitable. However, if cost of materials is correlated with $CO_2$ impact in the manufacturing stage, carbon anodes may still be a more acceptable choice in comparison with those inert anodes that are still under development. In fact, the more negative effect of using a carbon anode is carbon contamination of the cathodic products, which follows most likely two mechanisms as explained below.

### 5.1. Carbonate cycling mechanism

In the FFC Cambridge Process, the $CO_2$ gas produced on the carbon anode can be re-absorbed back into molten $CaCl_2$ and contaminate possibly the cathode product via reactions (17) and (18) [41,43–55]:

$$CO_2 + O^{2-} = CO_3^{2-}, \quad (CO_2 + CaO = CaCO_3) \qquad (17)$$

$$CO_3^{2-} + 4e = C + 3O^{2-} \qquad (18)$$

Reaction (18) was likely first reported in mid-1960 in molten carbonate salts [56–57], whereas later studies have suggested that this cathodic reaction can proceed in a wide range of molten salts as long as $CO_3^{2-}$ and $Li^+$ are present [18–21,56–60].

Thermodynamic analyses of cathodic deposition of carbon and alkali or alkaline earth metals revealed that $Ca^{2+}$ can also assist the cathodic deposition of carbon. This prediction was initially tested successfully in mixed $CaCl_2$ and $CaCO_3$ (84:16 molar ratio) [60], but the applied temperature (730°C) was higher than that of $Li^+$-containing carbonates (<600°C). The unique behavior of $Li^+$ and $Ca^{2+}$ (and $Ba^{2+}$) may have resulted from their high affinity to the $O^{2-}$ from reaction (18) (or the molten salt). This affinity pushes the metal deposition potential negatively away from that for carbon deposition. The same does not apply to $Na^+$ and $K^+$, leading to preference for metal deposition [60].

Unlike in molten carbonate salts or others with high $CO_3^{2-}$ activities, Reaction (18) in molten chloride salts is highly likely diffusion controlled. In addition, the fate of $CO_3^{2-}$ in molten $CaCl_2$ will depend on the experimental conditions. Thermal decomposition of $CaCO_3$ occurs at temperatures beyond 887°C, which will reduce (but unlikely eliminate) the $CO_3^{2-}$ activity. At a sufficiently high activity, $CO_3^{2-}$ may also compete with $O^{2-}$ to discharge on the carbon anode (reaction (19)) at a potential about 400 mV more positive than that of the reverse of reaction (18) [60].

$$2CO_3^{2-} + C = 3CO_2 + 4e \qquad (19)$$

Therefore, largely depending on the $O^{2-}$ activity in molten $CaCl_2$, the electro-reduction of $TiO_2$ does not necessarily always suffer from carbon contamination by reaction (18).

A systematic study of the products from electro-reduction of $TiO_2$ precursors (cylindrical pellets) with different porosities revealed an interesting trend of carbon contamination in products from low-porosity precursors but not in high-porosity ones [49,61]. Fig. 6(a) presents the XRD patterns of these products. The XRD patterns of the electrolyzed dense precursors were obtained from the samples' surface materials because the cores were partially or not reduced. In another experiment, $TiO_2$ precursors of the same porosity (70%) were electrolyzed in molten $CaCl_2$ with or without the added CaO. The products were analyzed by XRD (Fig. 6(b)). In both cases, carbon contamination was represented by the detection of TiC in the electrolysis products. Considering findings from both experiments, the TiC phase formed disregarding the porosity of $TiO_2$ precursors but appeared in products from electrolysis for longer times. In addition, as expected, the XRD patterns in Fig. 6(b) suggest convincingly a correlation of carbon contamination with the $O^{2-}$ activity in molten salts.

It was thought that the anodically generated $CO_2$ could travel to the cathode through both the gas and liquid phases. In the gas phase route, $CO_2$ enters the molten salt via reaction (17) at the gas/liquid interface, particularly near the cathode. In the liquid path, the as-produced $CO_2$ immediately reacts with $O^{2-}$ via reaction (17) in the molten salt near the anode and then transports to the cathode via convection and diffusion. Fig. 7(a) illustrates schematically this understanding. In this mechanism, both the time for mass transport of $O^{2-}$ and $CO_3^{2-}$ and the $O^{2-}$ activity for the conversion of $CO_2$ to $CO_3^{2-}$ play important roles.

### 5.2. Carbon debris mechanism

The other mechanism for carbon contamination is unique to the quality of commercial graphite used for making the anode. In laboratory studies of the FFC Cambridge Process, a thin layer of carbon debris is often observed on the surface of the molten salt. Such debris can also, depending on sizes, suspend in or precipitate to the bottom of the molten salt. In a scaling up test, a thick slug of carbon debris was observed on the top of molten $CaCl_2$ after electrolysis [62]. It is worth mentioning that various commercial graphite materials in the forms of rod and plate were used, but some of these materials performed better than the others in terms of stability, durability, and contamination to the molten salt. Because graphite crucibles are commonly used to contain molten salts without contamination, it is believed that the observation of carbon

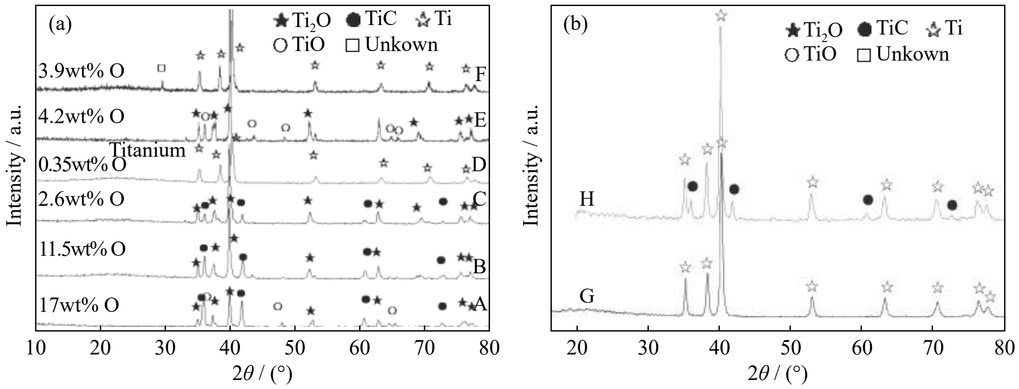

**Fig. 6. XRD patterns of products from electrolysis: (a) $TiO_2$ cylindrical pellets of different porosities (A: 12%, B: 23%, C: 44%, D: 68%, E: 77%, and F: 80%) at 3.2 V in molten $CaCl_2$ for 5 h (A, B, and C; surface materials) or 3 h (D, E, and F; full body); (b) $TiO_2$ pellets of 70% porosity in molten $CaCl_2$ at 3.0 V for 5 h (G), or in molten $CaCl_2$ + 5wt%CaO at 2.6 V for 11 h (H). Electrolysis temperature: 850°C [49].**

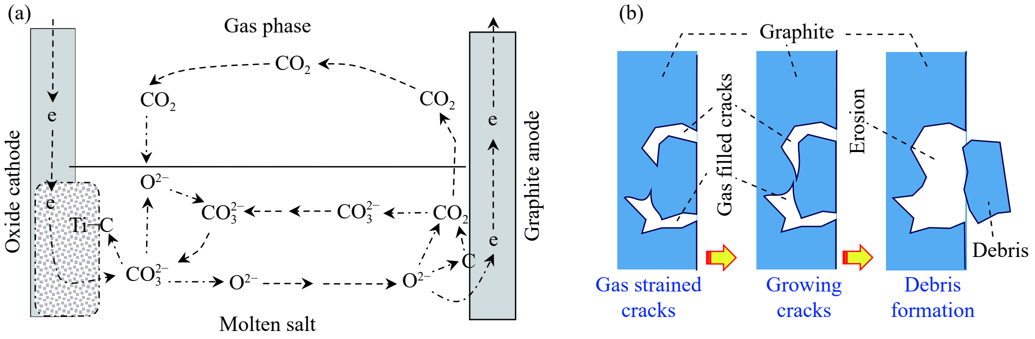

**Fig. 7. Schematic illustrations of two mechanisms of carbon transfer from the graphite anode to the oxide cathode during electrolysis of a solid metal oxide ($TiO_2$) in molten salts. (a) carbonate cycling mechanism: chemical and cyclic conversion between cathodically discharged $O^{2-}$, anodically formed $CO_2$, and chemically formed $CO_3^{2-}$ that is electro-reduced to carbon and contaminates the cathode. (b) Carbon debris mechanism: physical loss of carbon debris from the graphite anode by continuous formation of gas with an increasing pressure in cracks or pores that are either present originally in commercial graphite or formed by the loss of carbon at anodically selected sites for CO or $CO_2$ formation, leading to the eventual burst of confined gas and formation of carbon debris that floats on or suspends in the molten salt and eventually drifts to contaminate the cathode [12]. Reprinted from *Extractive Metallurgy of Titanium: Conventional and Recent Advances in Extraction and Production of Titanium Metal*, 1st ed, G.Z. Chen and D.J. Fray, Invention and fundamentals of the FFC Cambridge Process, 227, Copyright 2020, with permission from Elsevier.**

debris was related to the anodic behavior of graphite in $CaCl_2$-based molten salts.

This understanding agrees with experimental observations that graphite anodes were highly inert for discharging $S^{2-}$ to the $S_2$ steam or $Cl^-$ to the $Cl_2$ gas in the molten mixtures of chloride salts with low $O^{2-}$ solubility [32–35]. Specifically, carbon debris did not form in the molten mixture of $MgCl_2$, KCl, and NaCl, which prevented $O^{2-}$ from reaching the graphite anode by the formation of insoluble MgO and assisted in the successful electro-reduction of various metal oxides to the respective metals via anodic discharge of $Cl^-$ to $Cl_2$ [35].

Although no systematic study has been conducted on the formation mechanisms of carbon debris, two reasonable assumptions are worthy of discussion. First, the reduction of $CO_3^{2-}$, and likely directly $CO_2$, to carbon can proceed at the "gas/electrolyte/cathode current collector" 3PIs. The produced carbon may detach from the cathode and float as debris on the molten salt surface. However, this cathodic carbon formation mechanism may have only played a secondary role in the formation of carbon debris, if any. This is because dedicated cathodic deposition of carbon in molten carbonates has very high current efficiency [18–20,60], which means that most of the deposited carbon remained on the cathode upon collection.

Second, it was commonly noticed that the graphite anode was unevenly attacked or eroded after electrolysis (Fig. 1(h)). In other words, the oxidative attack is selective. The theoretical density of graphite is 2.26 g/cm$^3$, whereas commercial graphite rods and plates are manufactured by the densification of graphitic particles with densities commonly below 2.0 g/cm$^3$. Thus, commercial graphite has a certain level of porosity, with the pores, voids, or cracks existing more likely in the boundaries between packed particles. Additional surface pores or cracks on the anode surface can result from oxidative attack by oxygen atoms and molecules formed from $O^{2-}$ discharge, particularly to the boundaries. The pores or cracks on the surface of a graphite anode will be filled with molten salt where $CO_2$ forms. This process leads to two consequences. First, the pore deepens and widens due to carbon loss to $CO_2$ formation. Second, when the speed of $CO_2$ formation is faster than that of $CO_2$ escape from the pore, pressure builds up in the pores. Eventually, the increased $CO_2$ gas pressure in the pores and the weakened connection between graphite particles jointly force the detachment of carbon debris into the molten salt. Fig. 7(b) illustrates schematically this debris formation process on the carbon anode. Similar to the carbonate cycling mechanism, the carbon debris mechanism requires a certain time to proceed (to erode the anode gradually) and depends on the $O^{2-}$ activity in the molten salt (to enable $CO_2$ formation in pores and cracks).

### 5.3. Prevention of carbon contamination

The above discussion indicates that carbon contamination of the products in the FFC Cambridge Process originates from the use of a carbon anode. Therefore, as discussed above, the use of either an ionic blocking or conducting inert anode will eliminate this problem. However, inert anodes are still under development, making graphite a more preferable choice in the laboratory and industry. Furthermore, several approaches can help in reducing the negative influence of using a carbon anode on the cathode. The following sections discuss these approaches.

As mentioned above, the key step in the carbonate cycling mechanism is the reaction between $CO_2$ and $O^{2-}$ in the molten salt near the anode or at the gas/molten salt interface. However, the decomposition of $CaCO_3$ to CaO and $CO_2$ occurs at temperatures above 887°C. Thus, the carbonate cycling mechanism should be ineffective in molten $CaCl_2$ at high electrolysis temperatures [63]. Nevertheless, cautions should be applied when using this simple approach if the molten salts used contain other alkali and/or alkaline earth metal cations which can stabilize $CO_3^{2-}$ against decomposition. As shown by reactions (20) to (31), $CaCO_3$ decomposes, but $Na_2CO_3$ remains very stable at 950°C. Given that $CaCl_2$ has been often mixed with other chloride salts to lower the liquidus temperature, the carbonate cycling mechanism would not change in such melts by raising the electrolysis temperatures. The following reactions also include those for electroreduction of alkali or alkaline earth oxides against a carbon anode at 950°C. In these cases, $K_2O$ and $Na_2O$ are considerably easier to electrolyze than the other oxides because Na and K are gases, whereas all the other listed alkali and alkaline earth metals are liquids at 950°C.

$$MgCO_3 = MgO + CO_2,\ \Delta G^{\ominus}(950°C) = -105.2\ \text{kJ/mol} \tag{20}$$

$$2MgO + C = 2Mg + CO_2,\ \Delta G^{\ominus}(950C) = 539.4\ \text{kJ/mol} \tag{21}$$

$$CaCO_3 = CaO + CO_2,\ \Delta G^{\ominus}(950°C) = -9.1\ \text{kJ/mol} \tag{22}$$

$$2CaO + C = 2Ca + CO_2,\ \Delta G^{\ominus}(950°C) = 616.1\ \text{kJ/mol} \tag{23}$$

$$BaCO_3 = BaO + CO_2,\ \Delta G^{\ominus}(950°C) = 68.9\ \text{kJ/mol} \tag{24}$$

$$2BaO + C = 2Ba + CO_2,\ \Delta G^{\ominus}(950°C) = 480.1\ \text{kJ/mol} \tag{25}$$

$$Li_2CO_3 = Li_2O + CO_2,\ \Delta G^{\ominus}(950°C) = 53.6\ \text{kJ/mol} \tag{26}$$

$$2Li_2O + C = 4Li + CO_2,\ \Delta G^{\ominus}(950°C) = 568.2\ \text{kJ/mol} \tag{27}$$

$$Na_2CO_3 = Na_2O + CO_2,\ \Delta G^{\ominus}(950°C) = 149.8\ \text{kJ/mol} \tag{28}$$

$$2Na_2O + C = 4Na + CO_2,\ \Delta G^{\ominus}(950°C) = 83.5\ \text{kJ/mol} \tag{29}$$

$$K_2CO_3 = K_2O + CO_2,\ \Delta G^{\ominus}(950°C) = 211.8\ \text{kJ/mol} \tag{30}$$

$$2K_2O + C = 4K + CO_2,\ \Delta G^{\ominus}(950°C) = -51.7\ \text{kJ/mol} \tag{31}$$

Therefore, to avoid or reduce carbon contamination via the carbonate cycling mechanism, it is recommended to operate the FFC Cambridge Process in pure $CaCl_2$, with the $O^{2-}$ activity being as low as possible but still at a sufficient level to maintain a good cathodic reaction rate and the anodic discharge of $O^{2-}$ instead of $Cl^-$. The working temperature should be above 900°C but below 1000°C to avoid serious salt evaporation.

Physically reducing the direct contact between carbon debris and the oxide cathode was attempted to mitigate carbon contamination in the cathode. An alumina tube (sheath) was used to enclose the oxide pellet cathode from the gas phase and block most mass movement between the anode and cathode in the molten salt, leaving a small hole in the tube side wall to continue the ionic conduction path (Fig. 8(a)) [34,64–65]. This approach worked highly effectively in the elimination of the effect of carbon debris.

Figs. 8(b) and 8(c) compare the electrolysis products from the cell with and without using the alumina tube, respectively. Without using the tube, the as-electrolyzed cathode was covered by a solidified mixture of salt and carbon debris, whereas that electrolyzed inside the tube showed clear solidified salt only. In addition, using the tube in the electrolysis led to a significantly lower current flow than that without using the tube (Fig. 8(d)); however, the oxide pellet that was electro-reduced inside the tube could reach an oxygen content about 15wt% lower than that without using the tube [64]. These findings indicate high current and energy efficiencies. The efficiency derived from the electrolysis data in Fig. 8(d) was almost twice improved when using the alumina tube [65].

Such a significant improvement had benefited from the sheathing tube not only blocking electronic conduction through the floating carbon debris but also reducing the carbonate cycling. Whilst $CO_2$ transfer via the gas phase near the cathode was completely blocked, the route for $CO_3^{2-}$cycling through the molten salt was significantly narrowed by the small hole in the tube sidewall. The consequence would be

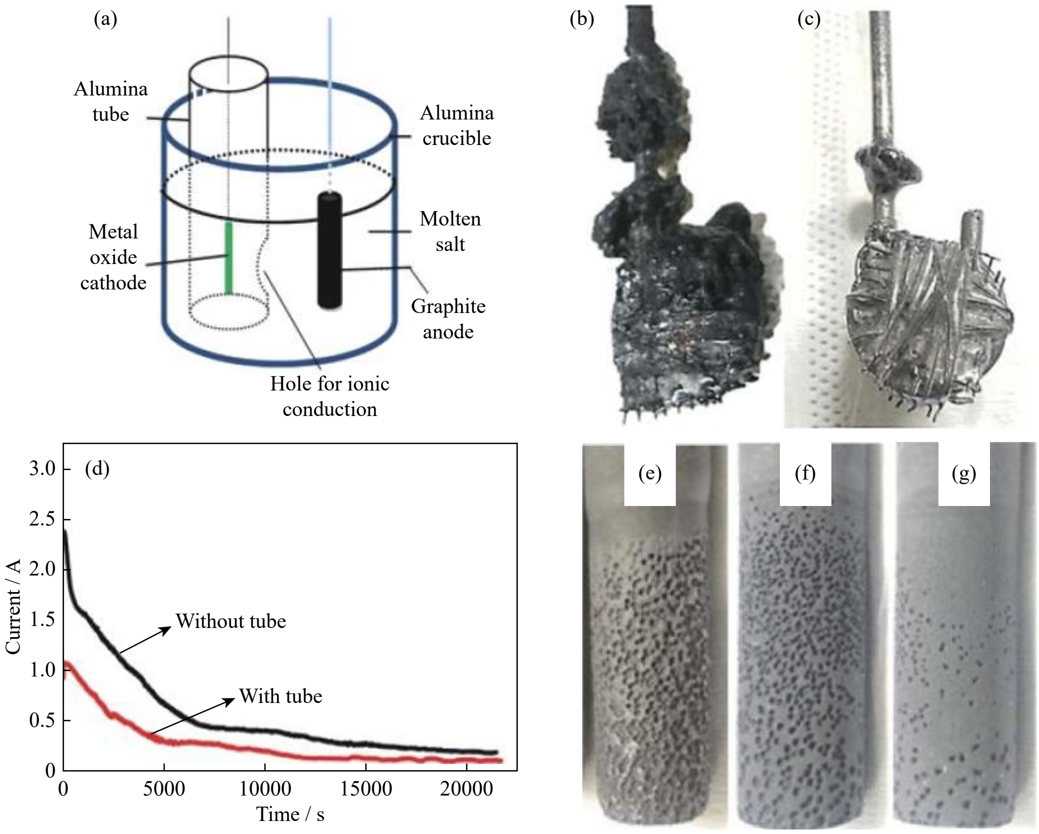

**Fig. 8. (a) Schematic of a modified FFC cell with a graphite rod anode and an oxide pellet cathode (1 g $Cr_2O_3$ wrapped by Mo wire), which was sheathed in an alumina tube whose internal and external molten salts were connected via a small hole on the alumina tube side wall facing the graphite anode. (b, c) Photographs of electrolyzed oxide cathodes without (b) and with (c) using the alumina sheath. (d) Current–time curves of electrolysis of $Cr_2O_3$ pellets at 2.80 V and 810°C without and with using the sheathing alumina tube. (e–g) Photographs of graphite anodes after electrolysis of $Cr_2O_3$ pellets without (e) and with (f, g) using the alumina tube ((f): frontside that was facing the hole during electrolysis; (g): backside) [64–65].**

that most $CO_3^{2-}$, if not all, in the molten salt were unable to reach the cathode in the same time interval but remained in molten salt outside the sheathing tube. This condition in turn reduced the amount of $O^{2-}$ produced by reaction (18) and hence the $O^{2-}$ activity and flux in the molten salt. This understanding was supported by the evident difference in anode erosion represented by the photographs in Figs. 8(e)–8(g).

Without using the sheathing tube, the graphite anode suffered from serious erosion and thinning (Fig. 8(e)), similar to that in Fig. 1(h). However, a remarkably less erosion occurred at the graphite anode (Figs. 8(f) and 8(g)) that was coupled with the alumina tube sheathed cathode. Erosion occurred around the graphite anode in absence of the sheathing tube, but it was more notable in the frontside facing the hole of the tube than on the backside (Figs. 8(f) and 8(g), respectively). These findings strongly prove that the $O^{2-}$ flux was smaller from the tube sheathed cathode than that from the naked cathode to the graphite anode, and such findings can be explained by the reduced $CO_3^{2-}$ cycling. In addition, given that the discharge of $O^{2-}$ is driven by the electric field in the interfacial layer between the electrode and electrolyte, i.e., the electric double layer, the difference between Figs. 8(f) and 8(g) is indicative of difference in the electric field strength. This result was less likely caused by the potential distribution around the graphite anode, which is a good electronic conductor. Instead, such findings can be attributed to the greater $O^{2-}$ activity and flux in front of the anode facing the hole of the sheathing tube. Expectedly, if the anode was placed at a sufficient distance from the tube hole, the frontside and backside of the anode would have behaved more similarly as diffusion and convection should even out the $O^{2-}$ distribution around the anode.

## 6. Other interactions

The above discussion focused on three main interactions that are common to all metal oxides to be reduced in the FFC Cambridge Process. However, several interactions are specific to the metal oxides or the metals produced. For example, $SiO_2$ can be electro-reduced in molten $CaCl_2$ to Si, which can react with Ca or $Ca^{2+}$ at appropriate cathodic potentials to form various calcium silicides, $CaSi_x$, with $x$ varying between 0.5 and 2.0 [66–68]. Reactions (32) and (33) below represent the chemical and electrochemical formation of $CaSi_x$, respectively, in which Ca may be assumed to result from the electro-reduction of $Ca^{2+}$ under certain depolarization conditions, e.g., on the surface of Si, although the actual origin of Ca is thermodynamically irrelevant.

$$Ca + xSi = CaSi_x,\quad (Ca + Si = CaSi,\ \Delta G^{\ominus}(850^{\circ}C) = -131.3\ kJ/mol) \tag{32}$$

$$xSiO_2 + (1+2x)Ca^{2+} + 2(1+2x)e = CaSi_x + 2xCaO,\quad (SiO_2 + Ca + C = CaSi + CO_2,\ \Delta G^{\ominus} = 181.5\ kJ/mol) \tag{33}$$

Because the formation of $CaSi_x$ occurs at potentials more positive than that for Ca deposition, the potential window for electroreduction of $SiO_2$ to pure Si is quite narrow [68]. Whilst a robust reference electrode [69–71] is needed to enable constant potential electrolysis in laboratory to avoid $CaSi_x$ formation, its application can be a great challenge in an industrial cell. The use of a computer-aided control (CAC) in a cell voltage program may offer a promising direction in industrial production [44]. This approach is simple, energy saving, and low cost. The principle of CAC is to program the cell voltage–time profile following what is pre-recorded in a successful test of constant-potential electrolysis.

Aluminum (Al) is difficult to produce from its oxide, $Al_2O_3$, in the FFC Cambridge Process, although thermodynamics can predict its feasibility as shown by reactions (34) and (35):

$$2/3Al_2O_3 + C = 4/3Al + CO_2,\quad \Delta G^{\ominus}(850^{\circ}C) = 484.4\ kJ/mol,\ \Delta E^{\ominus} = 1.255\ V \tag{34}$$

$$2CaO + C = 2Ca + CO_2,\quad \Delta G^{\ominus}(850^{\circ}C) = 638.8\ kJ/mol,\ \Delta E^{\ominus} = 1.655\ V \tag{35}$$

The difficulty is partly due to the relatively low melting point (660.3°C) of Al and the incapability of liquid Al to dissolve and transport oxygen. More importantly, the reduction potential of $Al_2O_3$ is only 0.40 V more positive than that for Ca deposition. Such a small difference can be easily overtaken by the polarization needed to reduce the highly insulating $Al_2O_3$, which indicates that the produced Al will react with Ca readily to form aluminides ($CaAl_x$, $x = 2, 4$). Similar to the case of Ca deposition on Si, the deposition of Ca on liquid Al can be depolarized and effectively form $CaAl_x$. The positive shift of the potential for Ca deposition on Al via reactions (36) and (37) can reach 1.032 and 1.008 V, respectively, which are greater than the difference between reactions (34) and (35).

$$Ca + 2Al = CaAl_2,\ \Delta G^{\ominus}(850^{\circ}C) = -199.1\ kJ/mol \tag{36}$$

$$Ca + 4Al = CaAl_4,\ \Delta G^{\ominus}(850^{\circ}C) = -194.5\ kJ/mol \tag{37}$$

The literature lacks sufficient information on the electro-reduction of solid $Al_2O_3$, although one report claimed the successful production of pure Al [72]; meanwhile, the others described the thermodynamic and experimental findings of $CaAl_x$ formation [73]. A recent study showed that when the $Al_2O_3$ powder was used on the cathode, electro-reduction produced $CaAl_2$, whereas pure Al beads were collected from the potentiostatic electrolysis of dense alumina (e.g., a small section of $Al_2O_3$ tube). Electrolysis of the small $Al_2O_3$ tube was assumed to favor the $O^{2-}$ diffusion away from the reaction site, i.e., the Al/$Al_2O_3$/electrolyte 3PIs, leaving behind a sufficiently low $O^{2-}$ activity (high $p_{O^{2-}}$ value) that helped production of pure Al as predicted by the thermodynamic analysis [74]. However, when electro-reducing the $Al_2O_3$ powder, diffusion in the pores between powder particles was too slow, leading to higher $O^{2-}$ activities to encourage formation, in analogy to perovskitization, of various calcium aluminates between $Ca_3Al_2O_6$ and $CaAl_{12}O_{19}$ ($xCaO\cdot yAl_2O_3$; $x$ = 1 to 3, $y$ = 1 to 6) whose electro-reduction can only produce $CaAl_x$. Obviously, the application of a constant cathodic potential was the key for the improved electrolysis result. The current industrial production of pure Al is achieved at a large scale by electrolysis of dissolved $Al_2O_3$ in molten fluoride salts with high rate and efficiency. Therefore, direct electro-reduction of solid $Al_2O_3$ to Al in molten chloride salts remains a fundamental interest.

As has been discussed above, the research on the FFC Cambridge Process in the past 20 years has succeeded in producing targeted metallic products owing to the relatively high stability of relevant metal oxides and sulfides in $CaCl_2$ (or LiCl)-based molten salts. However, the formation of oxychlorides can occur during electro-reduction of metal oxides to different extents. The awareness of oxychloride formation came from an early observation of a yellowish condensate [75] on the surface of the upper portions of a long graphite anode for electrolysis of $TiO_2$ in molten $CaCl_2$ (Fig. 1(f)). EDX analysis of the condensate indicated the presence of Ti, O, and Cl together with $CaCl_2$, which agrees with reaction (38). The Ti(III) valence exists in TiOCl, suggesting that the reaction has occurred upon partial reduction of the $TiO_2$ cathode. Reaction (38) is a side reaction, accounting for an insignificant percentage of the $TiO_2$ feed:

$$4TiO_2 + 2CaCl_2 + C = 4TiOCl + 2CaO + CO_2,\quad \Delta G^{\ominus}(900^{\circ}C) = 456.9\ kJ/mol \tag{38}$$

Most literatures on electro-reduction of metal oxides have neglected the formation of oxychloride, which can occur between most Re metal oxides and molten $CaCl_2$. Especially, lanthanum oxide ($La_2O_3$) reacts spontaneously with molten $CaCl_2$ to produce LaOCl, which may dissolve, at least partly, in molten $CaCl_2$.

$$La_2O_3 + CaCl_2 = 2LaOCl + CaO,\quad \Delta G^{\ominus}(850^{\circ}C) = -33.5\ kJ/mol \tag{39}$$

$$La_2O_3 + LiCl_2 = 2LaOCl + Li_2O,\quad \Delta G^{\ominus}(850^{\circ}C) = 40.8\ kJ/mol \tag{40}$$

Therefore, electro-reduction of $La_2O_3$ should not be carried out in molten $CaCl_2$, but it may still be feasible in molten LiCl. Nevertheless, the oxychloride issue can be avoided effectively by pre-compounding $La_2O_3$ with NiO [76]. In such a manner, various $AB_5$ hydrogen storage alloys (HSAs), such as $LaNi_5$ and $LaNi_4Co$, were prepared successfully by electroreduction in molten $CaCl_2$ at high yields, with the energy consumption being as low as 4.54 $kW \cdot h \cdot kg^{-1}$ for production of $LaNi_5$. After washing in water or DMSO, the as-produced HSAs all showed good charging–discharging performances. The obtained $LaNi_4Co$ powder performed most satisfactorily. A maximum discharging capacity of 325 $mA \cdot h \cdot g^{-1}$ was recorded, which is close to the theoretical capacity of 371.9 $mA \cdot h \cdot g^{-1}$ for $LaNi_5 + 3H_2 = LaNi_5H_2$.

Last, but not the least, in the FFC Cambridge Process, air-isolated high-temperature molten salts (e.g., $CaCl_2$, LiCl, and their mixtures with other salts) are used as the electrolyte. The applied high temperature helps in lowering the kinetic barriers for the electro-reduction of semiconducting and insulating solid metal oxides or sulfides to the respective metals. Air isolation, together with inert gas purging, is the most essential key to the success of the FFC Cambridge Process. To achieve air-isolation, it is crucial that the molten salt electrolyzer is strictly sealed in a reaction vessel. A general thought is that argon (Ar) is heavier than air, and can sink in the reaction vessel and protect the electrolyzer and cathode product from oxidation by the oxygen in air. However, such a thought is a misunderstanding and needs clarification. The explanation can be derived from the Gibbs energy changes of reactions (1) and (8), which show that the equilibrium partial pressures of oxygen are $2.53 \times 10^{-28}$ and $4.66 \times 10^{-34}$ Pa, respectively, whereas the oxygen partial pressure in air is $0.21 \times 10^5$ Pa. This ultra large difference in oxygen partial pressure means a huge diffusion rate of oxygen from air into the reaction vessel, even just through a small leaking gap.

Another misunderstanding is that the molten salt may provide a physical barrier for direct contact between the cathode product and air, hence protecting the former from oxidation. However, when $O^{2-}$ is present in a $CaCl_2$ or LiCl-based melt, which is inevitable in the FFC Cambridge Process, it can function as a phase transfer catalyst and transfer oxygen from the gas phase to the metal on cathode in the molten salt by the formation of peroxide ($O_3^{2-}$) and superoxide ($O_2^-$) anions via reactions (41) and (44), respectively [77].

$$O_2 + O^{2-} = O_3^{2-} \tag{41}$$

$$Ti + O_3^{2-} = TiO_2 + O^{2-} \tag{42}$$

$$O_2 + 2O_3^{2-} = 4O_2^- \tag{43}$$

$$3Ti + 4O_2^- = 3TiO_2 + 2O^{2-} \tag{44}$$

These reactions show that $O^{2-}$ transfers oxygen, as a vehicle offering a return service, from the gas phase to the Ti metal on cathode in the molten salt. After reaction with the metal, $O^{2-}$ is released back to the melt to transport more oxygen into the melt. Therefore, these two factors (i.e., oxygen diffusion in the gas phase due to leakage in the reaction vessel and $O^{2-}$ mediated oxygen transfer through the molten salt) will together make it impossible to electro-reduce a metal oxide to the metal with a sufficiently low oxygen content.

## 7. Summary

The FFC Cambridge Process has been in research and development for over two decades, embracing various successes but also challenges. Three generic interactions between the cathode product and molten salt, specifically $CaCl_2$, have been identified: (1) perovskitization and similar reactions that include Ca into the cathode without oxygen removal, (2) non-wetting of molten salts on pure metal surfaces, which helps in the separation of solidified salt in the final product, and (3) carbon contamination via either or both of the carbonate cycling and carbon debris mechanisms. In addition, observation and the mechanisms of formation of calcium silicides and calcium aluminides, formation of oxychlorides, and the problem of an unsealed or leaky reaction vessel were discussed briefly. In all the discussed cases of interactions, proper understanding of the causes and mechanisms of these interactions is the key to development and experimental demonstration of feasible approaches to desirable solutions.

In addition to the discussion on using porous oxide precursor to speed up electro-reduction, several studies were carried out on oxide pellets of extremely low porosity (≤30%), capturing more details of intermediate phases [78–80]. Another uncovered area of interest and technological importance is nuclear fuel processing [81–82], which deserves a separate specialist analysis. Further, the literature of the past two decades has included numerous comprehensive or specific topic review articles on the FFC Cambridge Process, all showing positive and encouraging views on prospects [6–12,39–50,68,83–85]. The literature has also provided scaling-up studies of the FFC Cambridge Process with promising results for eventual commercialization [13–14,84–86].

## Acknowledgements

This work was financially supported by the Darwin College Cambridge (Schlumberger Interdisciplinary Research Fellowship, 2000–2003), the Engineering and Physical Science Research Council of the UK (Nos. GR/J57650/02, GR/L08731/01, GR/S58447/01, EP/J000582/1, and EP/F026412/1), the Ministry of Education of China (2000–2003), the Natural Science Foundation of China (2002–2006), Zhejiang Provincial People's Government, China (2015–2019), and 3315 Talents Program from Ningbo Municipal People's Government, China (No. 2014A35001-1).

I would like to thank the editors of IJMMM for the invitation and patience in waiting for this manuscript, which has been written at a rather slow pace in the Covid-19 pandemic period following the UK policy of working from home. I fully appreciate the effort of the journal's language editor for proof reading and style-conversion of some original texts in the manuscript, in line with the journal's publishing tradition. I am sincerely grateful to my wife and children. Without their continuous understanding, support, and encouragement, pursuing my career in the research and development of the FFC Cambridge Process in the past two decades would have not been possible, particularly in the completion of this article in a locked down, uncertain, and depressing environment. I must express my great appreciation for the invaluable contribution from my past and present co-authors (whose names appear in the list of References) to the advancement of our understanding of the FFC Cambridge Process and of the relevant laboratory skills and equipment.

**Open Access** This article is licensed under a Creative Commons Attribution 4.0 International License, which permits use, sharing, adaptation, distribution and reproduction in any medium or format, as long as you give appropriate credit to the original author(s) and the source, provide a link to the Creative Commons licence, and indicate if changes were made. The images or other third party material in this article are included in the article's Creative Commons licence, unless indicated otherwise in a credit line to the material. If material is not included in the article's Creative Commons licence and your intended use is not permitted by statutory regulation or exceeds the permitted use, you will need to obtain permission directly from the copyright holder. To view a copy of this licence, visit http://creativecommons.org/licenses/by/4.0/.

## References

[1] D.J. Fray, T.W. Farthing, and Z. Chen, *Removal of Oxygen from Metal Oxides and Solid Solutions by Electrolysis in a Fused Salt*, International Patent, Appl. WO9964638, 1999.

[2] Chen GZ, Fray DJ, Farthing TW (2000). Direct electrochemical reduction of titanium dioxide to titanium in molten calcium chloride. Nature.

[3] Flower HM (2000). Materials Science: A moving oxygen story. Nature.

[4] Science and Technology, Dr. Chen and the philosopher’s stone, *The Economist*, 21st September 2000. [2020-05-4] https://www.economist.com/science-and-technology/2000/09/21/dr-chen-and-the-philosophers-stone

[5] Faller K, Froes FHS (2001). The use of titanium in family automobiles: Current trends. JOM.

[6] Fray DJ, Chen GZ (2004). Reduction of titanium and other metal oxides using electrodeoxidation. Mater. Sci. Technol..

[7] G.Z. Chen and D.J. Fray, Understanding the electro-reduction of metal oxides in molten salts, [in] A.T. Tabereaux ed., *Light Metals 2004*, Wiley-TMS, 2004, p. 881.

[8] Wang DH, Jin XB, Chen GZ (2008). Solid state reactions: An electrochemical approach in molten salts. Annu. Rep. Prog. Chem., Sect. C: Phys. Chem..

[9] Xiao W, Wang DH (2014). The electrochemical reduction processes of solid compounds in high temperature molten salts. Chem. Soc. Rev..

[10] Fray DJ, Schwandt C (2017). Aspects of the application of electrochemistry to the extraction of titanium and its applications. Mater. Trans..

[11] Hu D, Chen GZ, Breitkopf C, Swider-Lyons K (2017). Advanced extractive electrometallurgy. Springer Handbook of Electrochemical Energy.

[12] Chen GZ, Fray DJ, Fang ZZ, Froes HS, Zhang Y (2020). Invention and fundamentals of the FFC Cambridge Process. Extractive Metallurgy of Titanium: Conventional and Recent Advances in Extraction and Production of Titanium Metal.

[13] Metalysis, Technology, 2019 [2020-11-17]. http://www.metalysis.com/technology/.

[14] GLABAT, Development of Negative Electrode Materials, 2015 [2020-11-17]. http://www.glabat.com/article/content/view?id=23.

[15] Hu D, Xiao W, Chen GZ (2013). Near-net-shape production of hollow titanium alloy components via electrochemical reduction of metal oxide precursors in molten salts. Metall. Mater.Trans. B.

[16] Schwandt C, Hamilton JA, Fray DJ, Crawford IA (2012). The production of oxygen and metal from lunar regolith. Planetary Space Sci..

[17] B.A. Lomax, M. Conti, N. Khan, N.S. Bennett, A.Y. Ganin, and M.S. Symes, Proving the viability of an electrochemical process for the simultaneous extraction of oxygen and production of metal alloys from lunar regolith, *Planetary Space Sci.*, 180(2020), art. No. 104748.

[18] Siambun NJ, Mohamed H, Hu D, Jewell D, Beng YK, Chen GZ (2011). Utilisation of carbon dioxide for electro-carburisation of mild steel in molten carbonate salts. J. Electrochem. Soc..

[19] Tang DY, Yin HY, Mao XH, Xiao W, Wang DH (2013). Effects of applied voltage and temperature on the electrochemical production of carbon powders from CO_2_ in molten salt with an inert anode. Electrochim. Acta.

[20] W. Wang, B.M. Jiang, Z. Wang, and W. Xiao, In situ electrochemical conversion of CO_2_ in molten salts to advanced energy materials with reduced carbon emissions, *Sci. Adv.*, 6(2020), No. 9, art. No. 9278. 10.1126/sciadv.aay9278 PMC7048422 32158949

[21] O. Al-Juboori, F. Sher, A. Hazafa, M.K. Khan, and G.Z. Chen, The effect of variable operating parameters for hydrocarbon fuel formation from CO_2_ by molten salts electrolysis, *J. CO*_2_*Util.*, 40(2020), art. No. 101193.

[22] Peng C, Guan CZ, Lin J, Zhang SY, Bao HL, Wang Y, Xiao GP, Chen GZ, Wang JQ (2018). A rechargeable high-temperature molten salt iron-oxygen battery. ChemSusChm.

[23] Chen HL, Jin XB, Yu LP, Chen GZ (2014). Influences of graphite anode area on electrolysis of solid metal oxides in molten salts. J. Solid State Electrochem..

[24] Chen GZ, Fray DJ (2001). Cathodic refining in molten salts: Removal of oxygen, sulfur and selenium from static and flowing molten copper. J. Appl. Electrochem..

[25] Mohamedi M, Børresen B, Haarberg G, Tunold R (1999). Anodic behavior of carbon electrodes in CaO-CaCl_2_ melts at 1123 K. J. Electrochem. Soc..

[26] Chen GZ, Fray DJ (2002). Voltammetric studies of the oxygen-titanium binary system in molten calcium chloride. J. Electrochem. Soc..

[27] Barnett R, Kilby KT, Fray DJ (2009). Reduction of tantalum pentoxide using graphite and tin-oxide-based anodes via the FFC-Cambridge Process. Metall. Mater. Trans. B.

[28] Hu LW, Song Y, Ge JB, Jiao SQ, Cheng J (2016). Electrochemical metallurgy in CaCl_2_-CaO melts on the basis of TiO_2_RuO_2_ inert anode. J. Electrochem. Soc..

[29] Ramanarayanan TA, Rapp RA (1972). The diffusivity and solubility of oxygen in liquid tin and solid silver and the diffusivity. Metall. Trans..

[30] Zou XL, Lu XG, Zhou ZF, Li CH (2012). Direct electrosynthesis of Ti_5_Si_3_/TiC composites from their oxides/C precursors in molten calcium chloride. Electrochem. Commun..

[31] Hu HB, Gao YM, Lao YG, Qin QW, Li GQ, Chen GZ (2018). Yttria stabilized zirconia aided electrochemical investigation on ferric ions in mixed molten calcium and sodium chlorides. Mater. Metall. Trans. B.

[32] Wang T, Gao HP, Jin XB, Chen HL, Peng JJ, Chen GZ (2011). Electrolysis of solid metal sulfide to metal and sulfur in molten NaCl-KCl. Electrochem. Commum..

[33] Wu CH, Tan MS, Ye GZ, Fray DJ, Jin XB (2019). High-efficiency preparation of titanium through electrolysis of carbosulfurized titanium dioxide. ACS Sustainable Chem. Eng..

[34] Li W, Yuan YT, Jin XB, Chen HL, Chen GZ (2015). Environmental and energy gains from using molten magnesium-sodium-potassium chlorides for electro-metallisation of refractory metal oxides. Prog. Nat. Sci..

[35] Yuan YT, Li W, Chen HL, Wang ZY, Jin XB, Chen GZ (2016). Electrolysis of metal oxides in MgCl_2_ based molten salts with an inert graphite anode. Faraday Discuss..

[36] Sadoway D (2001). Inert anodes for the Hall-Heroult cell: The ultimate materials challenge. JOM.

[37] Yin HY, Gao LL, Zhu H, Mao XH, Gan FX, Wang DH (2011). On the development of metallic inert anode for molten CaCl_2_-CaO system. Electrochim. Acta.

[38] Yin HY, Tang DY, Zhu H, Zhang Y, Wang DH (2011). Production of iron and oxygen in molten K_2_CO_3_-Na_2_CO_3_ by electrochemically splitting Fe_2_O_3_ using a cost affordable inert anode. Electrochem. Commun..

[39] Wang DH, Xiao W, Lantelme F, Groult H (2013). Inert anode development for high-temperature molten salts. Molten Salts Chemistry-From Lab to Applications.

[40] Schwandt C, Fray DJ (2014). Use of molten salt fluxes and cathodic protection for preventing the oxidation of titanium at elevated temperatures. Metall. Mater. Trans. B.

[41] Schwandt C, Fray DJ (2005). Determination of the kinetic pathway in the electrochemical reduction of titanium dioxide in molten calcium chloride. Electrochim. Acta.

[42] Jiang K, Hu XH, Ma M, Wang DH, Qiu GH, Jin XB, Chen GZ (2006). “Perovskitization”-assisted electrochemical reduction of solid TiO_2_ in molten CaCl_2_. Angew. Chem. Int. Ed..

[43] Gordo E, Chen GZ, Fray DJ (2004). Toward optimisation of electrolytic reduction of solid chromium oxide to chromium powder in molten chloride salts. Electrochim. Acta.

[44] Wu T, Jin XB, Xiao W, Liu C, Wang DH, Chen GZ (2008). Computer-aided control of electrolysis of solid Nb_2_O_5_ in molten CaCl_2_. Phys. Chem. Chem. Phys..

[45] Chen GZ, Gordo E, Fray DJ (2004). Direct electrolytic preparation of chromium powder. Metall. Mater. Trans. B.

[46] Deng Y, Wang DH, Xiao W, Jin XB, Hu XH, Chen GZ (2005). Electrochemistry at conductor/insulator/electrolyte three-phase interlines: A thin layer model. J. Phys. Chem. B.

[47] Xiao W, Jin XB, Deng Y, Wang DH, Chen GZ (2007). Three-phase interlines electrochemically driven into insulator compounds: A penetration model and its verification by electroreduction of solid AgCl. Chem. Eur. J..

[48] Li W, Jin XB, Huang FL, Chen GZ (2010). Metal-to-oxide molar volume ratio: The overlooked barrier to solid-state electro-reduction and a green bypass through recyclable NH_4_HCO_3_. Angew. Chem. Int. Ed..

[49] W. Li, H.L. Chen, F.L. Huang, X.B. Jin, F.M. Xiao, and G.Z. Chen, Fast electro-reduction of TiO_2_ precursors with macro-micro-bimodal porosity in molten CaCl_2_, [in] *The 3rd Asia Conference on Molten Salts and Ionic Liquids*, Harbin, 2011.

[50] Chen GZ, Fray DJ (2006). A morphological study of the FFC chromium and titanium powders. Miner. Process. Extr. Metall..

[51] TWI Ltd., What is friction welding? [2020-07-04] https://www.twi-global.com/technical-knowledge/faqs/faq-what-is-friction-welding

[52] Muir Wood AJ, Copcutt RC, Chen GZ, Fray DJ (2003). Electrochemical fabrication of nickel manganese gallium alloy powder. Adv. Eng. Mater..

[53] Wang DH, Qiu GH, Jin XB, Hu XH, Chen GZ (2006). Electrochemical metallisation of solid terbium oxide. Angew. Chem. Int. Ed..

[54] Qiu GH, Wang DH, Ma M, Jin XB, Chen GZ (2006). Electrolytic synthesis of TbFe_2_ from Tb_4_O_7_ and Fe_2_O_3_ powders in molten CaCl_2_. J. Electroanal. Chem..

[55] Pistorius PC, Fray DJ (2006). Formation of silicon by electrodeoxidation, and implications for titanium metal production. J. South Afr. Inst. Min. Metall..

[56] Delimarskii YK, Gorodiskii OV, Grishchenko VF (1964). Cathode liberation of carbon from molten carbonates. Dokl. Akad. Nauk SSSR.

[57] Ingram MD, Baron B, Janz GJ (1966). The electrolytic deposition of carbon from fused carbonates. Electrochim. Acta.

[58] Kawamura H, Ito Y (2000). Electrodeposition of cohesive carbon films on aluminum in a LiCl-KCl-K_2_CO_3_ melt. J. Appl. Electrochem..

[59] Massot L, Chamelot P, Bouyer F, Taxil P (2003). Studies of carbon nucleation phenomena in molten alkaline fluoride media. Electrochim. Acta.

[60] Ijije HV, Lawrence RC, Siambun NJ, Jeong S, Jewell DA, Hu D, Chen GZ (2014). Electro-deposition and re-oxidation of carbon in carbonate containing molten salts. Faraday Discuss..

[62] K. Dring, Direct electrochemical production of titanium, [in] *The 3rd Workshop on Reactive Metal Processing (RMW3)*, Cambridge, 2007 [2020-07-09] http://www.okabe.iis.u-tokyo.ac.jp/core-to-core/rmw/RMW3/slide/RMW3_06_Dring_F.pdf.

[63] G.Z. Chen and D.J. Fray, *Prevention of Unwanted Reactions at Three Phase Boundaries*, UK Patent, Appl. GB0329541.7, 2003.

[64] Stevenson A (2016). Development of a Novel Electrochemical Pyro-processing Methodology for Spent Nuclear Fuels.

[65] Hu ML, Qu ZF, Bai CG, Hu D, Chen GZ (2017). Effect of the changed electrolytic cell on the current efficiency in FFC Cambridge Process. Mater. Trans..

[66] Xiao W, Jin XB, Deng Y, Wang DH, Chen GZ (2010). Rationalisation and optimisation of solid state electro-reduction of SiO_2_ to Si in molten CaCl_2_ in accordance with dynamic three-phase interlines based voltammetry. J. Electroanal. Chem..

[67] Yang X, Yasuda K, Nohira T, Hagiwara R, Homma T (2016). Cathodic potential dependence of electrochemical reduction of SiO_2_ granules in molten CaCl_2_. Metall. Mater. Trans. E.

[68] Juzeliūnas E, Fray DJ (2020). Silicon electrochemistry in molten salts. Chem. Rev..

[69] Gao P, Jin XB, Wang DH, Hu XH, Chen GZ (2005). A quartz sealed Ag/AgCl reference electrode for CaCl_2_ baeed molten salts. J. Electroanal. Chem..

[70] Wang H, Siambun NJ, Yu LP, Chen GZ (2012). A robust alumina membrane reference electrode for high temperature molten salts. J. Electrochem. Soc..

[71] Al-Shara NK, Sher F, Yaqoob A, Chen GZ (2020). Electrochemical investigation of novel reference electrode Ni/Ni(OH)_2_ in comparision with silver and platinum inert quasi-reference electrodes for electrolysis in eutectic molten hydroxide. Int. J. Hydrogen Energy.

[72] Xie HW, Zhang H, Zhai YC, Wang JX, Li CD (2009). Al preparation from solid Al_2_O_3_ by direct electrochemical deoxidation in molten CaCl_2_-NaCl at 550°C. J. Mater. Sci. Technol..

[73] Yan XY, Fray DJ (2009). Direct electrolytic reduction of solid alumina using molten calcium chloride-alkali chloride electrolytes. J. App. Electrochem..

[74] Kadowaki H, Katasho Y, Yasuda K, Nohira T (2018). Electrolytic reduction of solid Al_2_O_3_ to liquid Al in molten CaCl_2_. J. Electrochem. Soc..

[75] Clark RJH, Bradley DC, Thornton P (1973). The Chemistry of Titanium, Zirconium and Hafnium.

[76] Zhu Y, Wang DH, Ma M, Hu XH, Jin XB, Chen GZ (2007). More affordable electrolytic LaNi_5_-type hydrogen storage powders. Chem. Commum..

[77] Chen GZ, Fray DJ, Farthing TW (2001). Cathodic deoxygenation of the alpha-case on titanium and alloys in molten calcium chloride. Metall. Mater. Trans. B.

[78] Fray DJ (2008). Novel methods for the production of titanium. Int. Mater. Rev..

[79] Alexander DTL, Schwandt C, Fray DJ (2006). Microstructural kinetics of phase transformations during electrochemical reduction of titanium dioxide in molten calcium chloride. Acta Mater..

[80] Sri Maha Vishnu D, Sanil N, Shakila L, Panneerselvam G, Sudha R, Mohandas KS, Nagarajan K (2013). A study of the reaction pathways during electrochemical reduction of dense Nb_2_O_5_ pellets in molten CaCl_2_ medium. Electrochim. Acta.

[81] Choi EY, Lee JW, Park JJ, Hur JM, Kim JK, Jung KY, Jeong SM (2012). Electrochemical reduction behavior of a highly porous SIMFUEL particle in a LiCl molten salt. Chem. Eng. J..

[82] Stevenson A, Hu D, Chen GZ (2014). Molten salt assisted electrochemical separation of spent fuel surrogates by partial direct reduction and selective anodic dissolution. ECS Trans..

[83] Mohandas KS (2013). Direct electrochemical conversion of metal oxides to metal by molten salt electrolysis: A review. Miner. Process. Extra. Metall.

[84] Schwandt C, Doughty GR, Fray DJ (2010). The FFC-cambridge process for titanium metal winning. Key Eng. Mater..

[85] Hu D, Dolganov A, Ma MC, Bhattacharya B, Bishop M, Chen GZ (2018). Development of the fray-farthing-chen cambridge process: Towards the sustainable production of titanium and its alloys. JOM.

[86] Yu ZL, Wang N, Fang S, Qi XP, Gao ZF, Yang JY, Lu SG (2020). Pilot-plant production of high-performance silicon nanowires by molten salt electrolysis of silica. Ind. Eng. Chem. Res..

